# Determination of the Microbial Shift in the Gingival Sulcus of Women during Each Trimester of Pregnancy: A Cross-Sectional Study

**DOI:** 10.3390/medicina60101598

**Published:** 2024-09-28

**Authors:** Abdulaziz Alsakr, Ahmed Tawfig, Faisal Talal Almutairi, Ibrahim Mansour Ababtain, Hind Mohammed Saeed Alasmari, Banna Alnufaiy, Khalid Gufran

**Affiliations:** 1 Department of Preventive Dental Sciences, College of Dentistry, Prince Sattam bin Abdulaziz University, Alkharj 11942, Saudi Arabia; b.alnoufaiy@psau.edu.sa; 2 Department of Preventive Dentistry, College of Medicine and Dentistry, Riyadh Elm University, Riyadh 12734, Saudi Arabia; ahmamed.tawfig@riyadh.edu.sa; 3 Adan Specialized Dental Center, Department of Periodontology and Oral Medicine, Ministry of Health, Al-Adan 47402, Kuwait; ft.almutairi@moh.gov.kw; 4 General Directorate of Health Affairs in Riyadh Region, Department of Dentistry, Ministry of Health, Riyadh 12822, Saudi Arabia; imababtain@moh.gov.sa; 5 Department of Periodontics, Ministry of Health, Riyadh 13717, Saudi Arabia; alasmarihind@gmail.com

**Keywords:** dental plaque index, gingival crevice, gingival index, gram-positive, gram-negative, microbiota, pregnancy trimesters

## Abstract

*Background and Objectives*: This study aims to identify types of bacterial species in women visiting obstetrics/gynecology centers in Riyadh City, Saudi Arabia, during different pregnancy trimesters. *Materials and Methods*: This cross-sectional study was conducted among pregnant and nonpregnant women seeking care at Alyamamah Hospital, obstetrics/gynecology center, Riyadh, Saudi Arabia. A total of 110 [pregnant = 90 and nonpregnant = 20] individuals were recruited based on inclusion/exclusion criteria. Personal data, plaque index (PI), and gingival index (GI) were recorded. Bacterial samples were collected using sterile absorbent paper points from the gingival sulcus of pregnant females during the first, second, and third trimesters and compared to a control group of nonpregnant females. Thioglycolate broth containing the absorbent paper points was incubated at 37 °C for 24–48 h. After growth, the microorganisms were subjected to a Gram stain. The VITEK 2 system and conventional methods were used to identify various types of bacterial species from the gingival sulcus of pregnant and nonpregnant women. Chi-square tests and nonparametric tests were applied to the data. *Results*: The bacterial characterization indicated that *Actinomyces naeslundii* (AN) was the most predominant bacteria found in the study participants, followed by *Lactobacillus fermentum* (LF) (23.6%), *Veillonella* (VL) (10%), and unidentified organisms (9.1%). When the presence of subgingival bacterial species was compared between pregnant and nonpregnant women, a statistically significant difference was observed (*p* < 0.001). LF was the predominant bacteria in 9 nonpregnant women (45%) and 8 pregnant women in the first pregnancy trimester (44.4%). However, during the second (17 women: 48.6%) and third pregnancy (17 women: 45.9%) trimesters, AN becomes the predominant bacteria. A statistically significant difference was observed when the prevalence of various bacterial species was compared across the three pregnancy trimesters (*p* = 0.010). The plaque and gingival scores of pregnant and nonpregnant women showed no significant difference. *Conclusions*: In different trimesters of pregnancy, pregnant women’s gingival crevices showed significant microbial changes without affecting gingival inflammation.

## 1. Introduction

The Fédération Dentaire Internationale (FDI) recently redefined oral health as a multifaceted condition that includes the ability to speak, smile, smell, taste, touch, chew, swallow, and convey a variety of emotions through facial expressions with confidence and with the absence of pain, discomfort, or craniofacial-complex disease. The definition further states that oral health is a component of overall health, including physical and mental well- being [1]. The benefits of proper oral hygiene extend beyond avoiding dental caries and periodontal disease and include enhancing a person’s general state of health [2]. Multiple studies have shown a connection between a person’s overall systemic health and dental health [3,4].

The dental health of women must be given particular attention. Different physiological situations, including adolescence, pregnancy, and menopause, should be considered, since they affect women’s general health status [4]. Pregnant women’s dental health is paramount, since it directly affects the expectant mother and the unborn child’s future [5]. Pregnancy causes generalized changes in a woman’s body due to the progressive cycle of hormonal influences [6]. The most common conditions affecting periodontal health include gingivitis and periodontitis. It has been reported that at least 35% of pregnant women are usually affected by gingivitis [7]. Moreover, studies have shown ecological shifts in supragingival microbiota in pregnant women affecting the mother’s gingival health and the child’s growth and development [7,8]. 

Many pathogens may contribute to periodontal diseases, mainly gram-negative bacteria, including red, orange, and green complex bacteria. The red complex comprises *Porphyromans gingivalis*, *Tannerella forsythia*, and *Treponema denticola*. In comparison, the orange complex consists of *Prevotella intermedia* and *Fusobacterium nucleatum*. The green complex consists of Eikenella *corrodens* and *Capnocytophage* species, with *Aggregatibacter Actinomycetemcomitans* in a separate category (purple) [9].

Researchers found that clinical periodontal diagnosis positively correlated with quantifying the sub-gingival microbiota at different trimesters of pregnancy. Moreover, it was observed that the microbial species *Tanerella forsythia* was common during the first trimester of pregnancy, but its abundance decreased significantly toward the third trimester [10,11]. Sex hormones can influence or alter the oral microbiome’s compositions, leading to shifts in immune responses and dysbiosis. As a result, periodontal infections in pregnant women may cause severe systematic gingival inflammatory responses. Further, it was noted that factors such as low birth weight, pre-eclampsia, other pregnancy complications, activation of the maternal inflammatory cell responses, cytokine release, and dysbiosis in the oral microbiota play a possible role in causing gingival complexities during different trimesters of pregnancy [12]. During pregnancy, the oral microbiome undergoes a pathogenic change that reverts to a baseline or “healthy microbiome” during the postpartum period, with female sex hormones such as progesterone and estrogen thought to mediate this shift [13,14].

To improve prediction and intervention strategies for unfavorable pregnancy outcomes, it is essential to comprehend the oral microbiota alterations that occur throughout pregnancy and their relationship with adverse pregnancy outcomes. Despite the studies on oral microbiota shifts during pregnancy trimesters, there is no agreement about the true nature of microbiome changes during pregnancy, since contradictory and surprising data have been reported. Hence, the present study is required to understand how the gingival microbiota shifts occur at various stages of pregnancy. Thus, this study aims to identify different types of bacterial species from gingival sulcus during different pregnancy trimesters in women visiting obstetrics/gynecology centers in Riyadh City, Saudi Arabia.

## 2. Materials and Methods

This study was conducted among pregnant women attending the gynecologic/obstetric center at Alyamamah Hospital, Riyadh, Saudi Arabia. All the study participants underwent a clinical periodontal examination and gingival crevicular fluid sampling in the first, second, and third trimesters with a control group of nonpregnant ladies.

The study proposal was submitted to the research and innovation center of Riyadh Elm University, and approval was obtained (FPGRP/2021/645/693/680). Alyamamah Hospital, Riyadh authorities were contacted, and a written agreement was made to conduct the study. Before the investigation, the purpose of the study was described to the participants, and signed informed consent was acquired. 

The sample size was calculated by compromise power analysis in G* Power considering an effect size of (Chi-Square tests) 0.5, alpha error probability of 0.05, and power of the study 0.95. It resulted in a sample size of 109, which was adjusted to the nearest number of 110. Ninety out of the total were pregnant, and 20 were nonpregnant women.

The study included patients who are Saudi nationals and pregnant women in all trimesters. Patients with systemic diseases and young women under the age of 15 were excluded from the study. The study excluded individuals receiving corticosteroid therapy and heparin, those who had undergone systemic antibiotics in the previous four weeks, and patients with deep periodontal pockets.

Data collection: A ten-item questionnaire derived from previous research [6] and clinical oral examination was used to collect primary data. The questionnaire briefly described the study, an invitation to participate, and a consent form to be signed. The questionnaire comprised two sections. Section one enquired about the socioeconomic background (age and education). Section two recorded practices related to gingival health: tooth-brushing frequency (No brushing, Once/day or more), smoking (yes/no/in the past), daily use of sugary drinks and sugary foods (yes/no), and pregnant frequency (no pregnancy, once or more).

The clinical examination: A single-trained periodontic resident performed all oral examinations by strictly following an infection control protocol while recording plaque and gingival index scores. Dental plaque was recorded using Silness and Loe’s (1964) [15] plaque index, and the gingival index was provided by Loe and Silness (1963) [16].

Microbiological Analysis: Samples’ collection and bacterial isolation: The sample collection and clinical examination were completed on the same day, and the samples were immediately sent to the laboratory for incubation. Samples were collected using sterile absorbent paper points (META BIOMED-KOREA) size 30 (Guentsch et al. 2011) [17] from the gingival sulcus of the mesial side of the maxillary right second premolar (Kornman 1980) [10] of pregnant females during the first, second, and third trimesters, along with a control group of nonpregnant females. The sterile absorbent paper points were placed in the gingival sulcus until light pressure was felt for thirty seconds (Bunaes et al. 2017) [18] and then transferred to thioglycolate broth used to grow anaerobic bacteria. Thioglycolate broth containing the absorbent paper points was incubated at 37 °C for 24–48 h. After growth, the microorganisms were subjected to Gram staining to differentiate Gram-positive and Gram-negative bacteria. Subsequently, the subculture was performed using a sheep blood agar plate with anaerobic gas using an anaerobic jar Kit (BR0038) Thermo Fisher Scientific (Waltham, MA USA)] and was transferred into an incubator at 37 °C for 24–48 h.

Bacterial identification: The conventional identification of the bacterial species was carried out by an experienced microbiologist from the department of basic sciences at Riyadh Elm University, Riyadh, Saudi Arabia. In addition, the VITEK 2 system (Biomerieux-Durham, NC, USA) was used for fast and accurate microbial identification. Based on the VITEK 2 system, bacterial species were identified in Anfas Alraha’s private laboratory in Riyadh city, Saudi Arabia. The VITEK 2 system identified various types of bacterial species from the gingival sulcus of pregnant women at different trimesters of pregnancy and among nonpregnant women. The VITEK 2 system offers an extensive identification and susceptibility menu and an expanded identification database, and it is the most automated platform available.

Reagent Cards used with VITEK 2 machine: The reagent cards have 64 wells that can each contain an individual test substrate. Substrates measure various metabolic activities, such as acidification, alkalinization, enzyme hydrolysis, and growth in the presence of inhibitory substances. For inoculation, each card has a pre-inserted transfer tube. The card’s bar codes contain information on product type, lot number, expiration date, and a unique identifier, which can be associated with the sample before or after loading the card into the system. There are currently four reagent cards available for identifying different organism classes, which are as follows: GN: Gram-negative fermenting and non-fermenting bacilli; GP: Gram-positive cocci and non-spore-forming bacilli; YST: Yeasts and yeast-like organisms; and BCL: Gram-positive spore-forming bacilli.

Culture Required: The culture of bacteria is needed according to the organisms isolated, such as blood Agar, Columbia blood agar, neomycin blood agar, etc.

Suspension Preparation: Transfer a sufficient number of colonies of a pure culture using a sterile swab or applicator stick, then suspend the microorganism in 3.0 mL of sterile saline (aqueous 0.45% to 0.50% NaCl, pH 4.5 to 7.0) in a 12 mm × 75 mm clear plastic tub.

Inoculation: An integrated vacuum apparatus inoculates identification cards with microorganism suspensions. Place a test tube containing the microorganism suspension into a cassette rack, insert the transfer tube into the corresponding suspension tube, and place the identification card in the corresponding slot. Once you apply the vacuum and reintroduce air into the station, the transfer tube forces the organism suspension into micro-channels, filling all the test wells.

Card Sealing and Incubation: A mechanism passes inoculated cards, cutting off the transfer tube and sealing the card before loading it into the carousel incubator. The carousel incubator can store up to 30 or 60 cards. All card types are incubated online at 35.5 + 1.0 °C (8 to 12 h).

Test Reactions: Calculations are performed on raw data and compared to thresholds to determine reactions for each test. On the VITEK 2 Compact, test reaction results appear as “+”, “−”, “(–)” or “(+)”. Reactions that appear in parentheses are indicative of weak reactions that are too close to the test threshold.

Database Development: Large strain sets of well-characterized microorganisms tested under various culture conditions form the databases for the VITEK 2 identification products. These strains are derived from a variety of clinical and industrial sources as well as from public (e.g., ATCC) and university culture collections. The bacterial species identified are shown in Table 1.

Statistical analysis: Descriptive statistics of frequency distribution and percentages were calculated for the categorical variables (personal characteristics, oral health-related variables, and bacterial species). The mean score/ranks and standard deviation values were obtained for continuous variables (plaque and gingival scores). Chi-square and Fisher’s exact tests were applied to assess the relationship between pregnancy status and personal characteristics, different bacterial species in pregnant and nonpregnant women, bacterial species in nonpregnant and in different pregnancy trimesters, different bacterial species and pregnancy frequencies, different pregnancy trimesters and Gram stain bacteria, pregnancy frequencies and Gram stain bacteria, and Gram stain bacteria in nonpregnant and pregnant women. Normality tests indicated a non-normal distribution of the PI and GI scores. Hence, a nonparametric Mann–Whitney U test was applied to compare mean PI and GI score/ranks between pregnant and nonpregnant women, while Kruskal–Wallis’s test was applied to compare mean PI and GI score/ranks across pregnancy frequencies, pregnancy trimesters and different bacterial types. A value of *p* < 0.05 was considered significant for all the statistical tests. All the data were analyzed using a statistical analysis package (IBM-SPSS version 25, Armonk, NY, USA).

## 3. Results

A total of 110 women (*n* = 110) visiting Alyamamah hospital gynecology/obstetric center participated in this study. The characteristics of the study participants are shown in Table 2.

Of the 110 study participants, 90 (81.8%) were pregnant and 20 (18.2%) were non-pregnant. The majority of participants, 96 (87.3%), were in the 26–40 age group, 107 (97.3%) weighed 61–100 kg, and 105 measured 140–180 cm in height (95.5%). More than half of the study participants had a university level of education (64: 58.2%), 74 (67.3%) avoided sugar drinks, and none were smokers. The oral health-related variables indicated that most of the study participants brushed their teeth once per day (73: 66.4%) and that 87 (79.1%) did not perform oral disinfection. The reported mean ± SD values of plaque and gingival scores were 1.59 ± 0.63 and 1.68 ± 0.60, respectively.

The pregnancy-related variables and the bacterial species characterized in this study are shown in Table 3. More than half of the study participants had two or more pregnancies.

**Figure 1:**
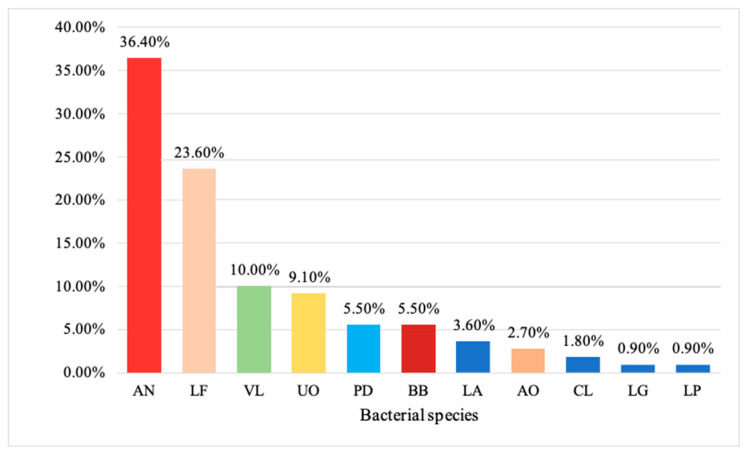
Different bacterial species observed among study participants (*n* = 110).

Nearly 65 (59.1%) and 25 (22.7%) participants were first-time pregnant. Most of the study participants were in their third trimester of pregnancy (37: 33.6%), followed by the second (35: 31.8%) and first trimester (18: 16.4%). The bacterial characterization indicated that *Actinomyces naeslundii* (AN) was the most predominant bacteria found in the study participants, followed by *Lactobacillus fermentum* (LF) (23.6%), *Veillonella* (VL) (10%) and *Unidentified organisms* (9.1%), while *Lactobacillus plantarum* (LP) (0.9%) was the least prevalent bacteria in the study sample (Figure 1). The majority of the bacteria observed in the study sample were Gram-positive (83: 75.5%).

The relationship between pregnancy status and the personal characteristics of the study participants is shown in Table 4. The pregnant and nonpregnant women did not differ significantly in age (*p* = 0.281), weight (*p* = 0.408), height (*p* = 0.195), and sugar drink intake (*p* = 0.196). However, pregnant and nonpregnant women differed significantly across educational levels (*p* = 0.029) and tooth-brushing frequency (*p* < 0.001).

**Figure 2:**
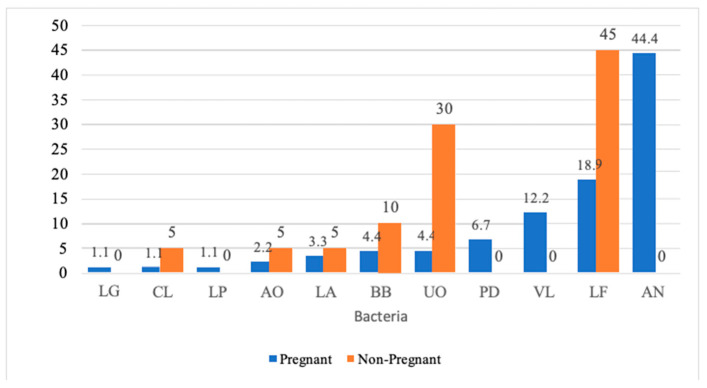
Distribution of different bacterial species between pregnant and nonpregnant study participants.

Pregnancy and gingival sulcular microbiota: The characterization of subgingival bacterial species of pregnant and nonpregnant women is shown in Figure 2. AN 40 (36.4%) was the predominantly observed bacterial species (as shown in Table 5) among the studied sample. The pregnant women’s subgingival sulcus predominantly demonstrated AN (40: 44.4%), followed by LF (17: 18.9%), VL (11: 12.2%), PD (6: 6.7%), BB (4: 4.4%), UO (4: 4.4%), LA (3: 3.3%), LG (1: 1.1%), CL (1: 1.1%) and LP (1: 1.1%). In contrast, nonpregnant women showed mainly LF (9: 45%), followed by UO (6: 30%), BB (2: 10%), LA (1: 5%), AO (1: 5%), and CL (1: 5%). However, LG, AN, PD and VL were absent in the gingival sulcus of nonpregnant women. When the presence of subgingival bacterial species was compared between pregnant and nonpregnant women, a statistically significant difference was observed (*p* < 0.001).

**Figure 3:**
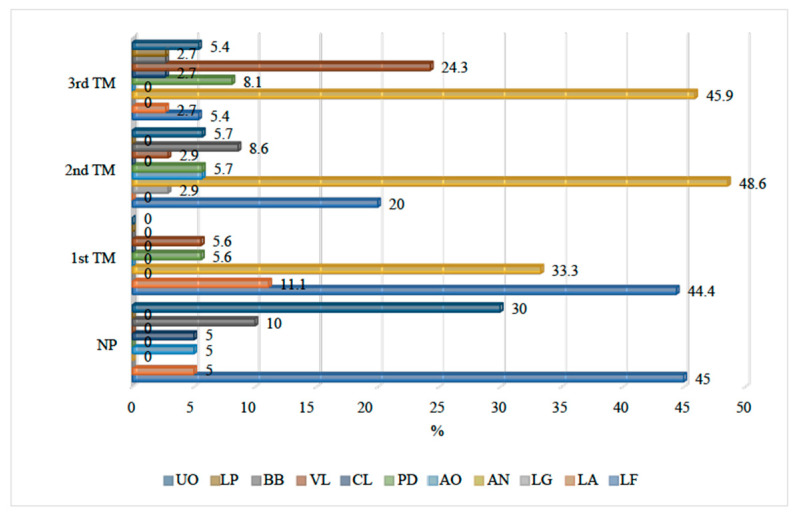
Bacterial species in nonpregnant women and in different pregnancy trimesters.

The bacterial species in nonpregnancy and during the different pregnancy trimesters is shown in Figure 3. LF was the predominant bacteria in nonpregnant (9: 45%) women and pregnant women in the first pregnancy trimester (8: 44.4%). However, AN became the predominant bacteria during the second (17: 48.6%) and third pregnancy (17: 45.9%) trimesters.

The following are the bacterial species found across various trimesters: LF 8 (44.4%) is the most abundantly found bacterial species during the first trimester of the pregnancy, followed by AN (6: 33.3%) and others. In comparison, AN (17: 48.6%) and LF (7: 20%) were commonly found during the second trimester of pregnancy, while AN (17: 45.9%) and VL (9: 24.3%) were the most prevalent bacterial species observed in the third trimester of the pregnancy. A statistically significant difference was observed when the prevalence of various bacterial species was compared across the three pregnancy trimesters (*p* = 0.010).

The relationship between different bacterial species and pregnancy frequencies is as follows: The bacterial species AN was large in first-time pregnant women (15: 60%) and in those with two or more pregnancies (25: 38.5%). However, bacterial species LG, CL, BB, and LP were absent in first pregnancies compared to those with two or more pregnancies. The presence of different bacteria did not differ significantly between first-time pregnant women and those with two or more pregnancies (*p* = 0.568).

The distribution of Gram stain bacteria in the different trimesters of pregnancy was examined. We observed a large proportion of Gram’s positive bacteria in the first trimester (16: 88.90%), second trimester (30: 85.70%), and third trimester (23: 62.20%). On the other hand, we reported Gram’s negative bacteria in the first (2: 11.10%), second (3: 8.60%), and third (14: 37.5%) trimesters of pregnancy. In addition, 2 (5.70%) unknown bacteria were found in the second trimester of pregnancy. When sulcular bacterial species were assessed based on Gram’s staining across the three pregnancy trimesters, a statistically significant difference was observed (*p* = 0.007).

The distribution of Gram stain bacteria was observed in first-time pregnant women and in those with two or more pregnancies. Gram-positive bacteria were predominant in first-time pregnant women (21: 84%) and in those with two or more pregnancies (48: 73.8%). In contrast, Gram-negative bacteria numbered 3 (12%) and 16 (24.6%) in first-time pregnant women and in those with two or more pregnancies. A minor percentage of unknown bacteria was observed in both groups. A comparison of bacteria based on Gram stain showed no significant difference between the first-time pregnancy and two or more pregnancies (*p* = 0.260).

### Clinical Parameters and Pregnancy

The comparison of PI scores between pregnant (1.59 ± 0.60) and nonpregnant women (1.60 ± 0.75) showed no significant difference (*p* = 0.900). Similarly, GI between pregnant (1.67 ± 0.58) and nonpregnant women (1.75 ± 0.72) showed no significant difference (*p* = 0.710). The mean PI and GI scores did not differ significantly across pregnancy frequency (*p* = 0.244, *p* = 0.443) and pregnancy trimesters (*p* = 0.256, *p* = 0.392). PI varied significantly across different bacterial species (*p* = 0.036). However, no significant difference was observed with the GI score (*p* = 0.378).

## 4. Discussion

The primary purpose of this research was to make a qualitative assessment of gingival sulcular microbiota in terms of bacterial species or type during different pregnancy trimesters. Secondly, it was to identify the microbial shift occurring in different trimesters of pregnancy compared to nonpregnant women. It has been reported that different types of organisms were identified in all the samples from pregnant and nonpregnant women [10]. 

In the present study, bacterial species were compared between pregnant and non-pregnant women, and a statistically significant difference was observed. Specific strains of LG, AN, PD and VL were found only in pregnant women compared to nonpregnant women. Hence, the null-hypothesis according to which there would be no microbial shifting in the gingival sulcus occur during different pregnancy trimesters has been rejected.

In this study, the gingival sulcus of pregnant women demonstrated a remarkable increase in AN. However, previous studies have reported an increase in the proportion of Bacteroides intermedius, *Clostridium bifermentans* in the gingival sulcus of pregnant women [10,19]. Haffajee et al. reported that there were differences in the prevalence of subgingival bacterial species by country; for instance, *Prevotella melaninogenica* was identified in about 6% of Chilean and Swedish patients but not in subjects from other countries. Also, *Veillonella parvula* was detected at a higher percentage among Americans in comparison to Swedish subjects [20,21]. Another study reported subgingival bacterial profile changes over the course of pregnancy as well as after delivery [22]. 

Moreover, AN continues to be the significantly predominant bacteria in second and third trimesters of pregnancy compared to the first trimester of pregnancy. In contrast, LF was predominantly observed in the gingival sulcus of nonpregnant and pregnant women in the first trimester of pregnancy. Thus, a clear microbial shift was observed from the first trimester of pregnancy to the second and third trimesters. In addition, when bacterial species were compared between first-time pregnancy and two or more pregnancies, AN was found to be the predominant bacteria, with no significant difference. It is speculated that the microbial shift could be attributed to the hormonal variations observed during the pregnancy, since previous studies have indicated that pregnancy hormones seem to be capable of altering the normal subgingival bacterial flora and subgingival ecology [23,24,25,26,27]. 

In our study, three-fourths of the study participants demonstrated the presence of gram-positive (LF, LA, LG, AN, AO, CL, BB and LP) bacterial species. As the pregnancy progressed from the first trimester to the third trimester, gram-positive species were replaced by gram-negative (PD and VL) species. This bacterial transition was statistically significant, with the presence of some unknown bacterial species. This finding can be corroborated with the study by Kornman and Loesche (1980) [10], who reported that gram-negative anaerobic rods accounted for approximately 10% of the total flora during the first trimester. Gram-negative anaerobic rods had increased to 39% of the flora during the second trimester of pregnancy.

The majority of studies, however, focused on what Socransky et al. called the orange complex (*Peptostreptococcus micros*, *Prevotella nigrescence*, *Fusobacterium nucleatum*, and *Prevotella intermedia*) and the “red complex” (*Campylobacter rectus*, *Tannerella forsythia*, *Treponema denticola*, and *Porphyromonas gingivalis*) [9,28]. These organisms may be entirely missing from the research participants, or Vitek 2 may have overlooked them in error (unknown organisms). Gram-positive and gram-negative bacterial species did not differ significantly across pregnancies frequencies.

In this study, the plaque score did not show any significant difference between the pregnant and nonpregnant participants. The plaque scores gradually increased as the pregnancy trimester progressed, with the highest plaque score being observed during the third trimester. When plaque scores were compared across different pregnancy trimesters and nonpregnant women, there was no significant difference found. This finding is in accordance with the studies that reported a gradual increase in the plaque score in the first, second and third trimester of pregnancy [29]. In contrast, a few studies observed no fluctuation in plaque scores throughout pregnancy and in non-pregnant women [10,15]. It can be speculated that the free prenatal dental counselling, educational level and self-motivation of pregnant women could have contributed to better oral hygiene behavior and the insignificant differences in plaque scores. Similarly, women with ≥2 pregnancies showed a high plaque score, which did not differ significantly across first-time pregnant and nonpregnant women. In addition, plaque scores demonstrated a significant difference across the presence of bacterial species. The study participants showed the highest plaque scores in the presence of LG and CL, while the lowest score was found with AO and PD.

The present study has shown varying degrees of gingival inflammation between pregnant and nonpregnant women, with an insignificant difference. Similarly, gingival score varied across nonpregnant women and pregnant women in different trimesters, with a gradual increase in the gingival scores. In line with this study, Jonsson et al. (1988) found no significant difference in the gingival health of pregnant and post-partum women [30]. In contrast to this study, several reports have shown a statistically significant difference in gingival inflammation between nonpregnant and pregnant women in different trimesters [10,12,14,15,16,31]. The variation in the observed gingival score could be due to the fact that the prevalence, extent and severity of gingival inflammation during pregnancy differ considerably among various reported studies. Methodological heterogeneity may, at least in part, explain differences in the obtained results. Cross-sectional studies [15,16,30,32] in comparison to longitudinal studies [29,31,33] hamper the analysis of the relationship between pregnancy and the exacerbation of gingival inflammation [32,33,34]. Other factors that vary within different research groups may have affected the wide range of results obtained, including the use of different clinical indices, study designs, measurement equipment and the control of confounding factors [35]. 

### Strengths & Limitations of the Study

This is the first study carried out in Riyadh, and not many are conducted around the world. The subjects in the pregnant and nonpregnant groups were recruited from one single health-care center, and they were assumed to be homogenous in their ethnicity and socio-economic status. Since younger or older women may have hormonal fluctuations other than pregnancy, such as puberty or menopause, we limited the age range of the present study participants to 18–40 years. Limiting the age range prevented these variables from potentially influencing the periodontal condition of the pregnant study population, thereby enhancing the strength of the results.

Even though the study demonstrated positive strengths, the current study possessed some limitations. The study was restricted to the assessment of the gingival crevicular microbiota and clinical indices on plaque and gingival inflammation. Hence, other periodontal parameters, such as pocket depth, alveolar bone loss, gingival biotype and clinical attachment levels, have not been recorded. No attempt was made to estimate salivary, blood or tissue concentrations of the hormone levels, especially estrogen and progesterone. The study sample included a large number of pregnant women and only a few nonpregnant subjects. Despite the limitations, the present study provided an insight into the gingival crevice microbiota in pregnant women.

Future longitudinal follow-up studies of pregnant women, with a large sample size based in multiple centers, are required to confirm the current study findings. An estimation and correlation of the hormonal levels, gingival sulcular microbiota, and clinical periodontal parameters, including gingival biotype, are required to establish an objective relationship between variables.

## 5. Conclusions

*Actinomycetes naeslundi* was the predominant bacteria observed in the study. Lactobacillus fermentum is the most common bacteria found in nonpregnant women and pregnant women during the first trimester of pregnancy. However, *Actinomycetes naeslundi* was the main bacterial species found in the pregnant women during the second and third trimesters of pregnancy. 

During pregnancy, we observed a significant shift in the bacterial microbiota, potentially due to hormonal changes. Moreover, *Actinomycetes naeslundi* remains the main bacterial species, regardless of pregnancy frequency (first-time pregnancy or two or more pregnancies). All the pregnancy trimesters observed significant changes in gram-positive and gram-negative bacteria. Different trimesters of pregnancy revealed a notable microbial shift in the gingival crevices of pregnant women, without any impact on gingival inflammation.

## Figures and Tables

**Table 1:** Bacterial species identified in this study.

Name of the Bacteria	Abbreviation
*Lactobacillus fermentum*	LF
*Lactobacillus acidophilus*	LA
*Lactobacillus gasseri*	LG
*Actinomyces naeslundii*	AN
*Actinomyces odontolyticus*	AO
*Parabacteroides distasonis*	PD
Clostridium group	CL
*Veillonella*	VL
*Bifidobacterium* spp.	BB
*Lactobacillus plantrarum*	LP
*Unidentified organism*	UO

**Table 2:** Characteristics of the study participants (*n* = 110).

Variables		*n*	%
1. Personal Characteristics			
Status	Pregnant	90	81.8%
	Nonpregnant	20	18.2%
Age (Years)	18–25	14	12.7%
	26–40	96	87.3%
Weight (Kilograms)	<60	3	2.7%
	61–100	107	97.3%
Height (Centimeters)	140–180	105	95.5%
	180–200	5	4.5%
Education	School	46	41.8%
	University	64	58.2%
Smoking	Yes	0	0.0%
	No	110	100.0%
Sugar drinks	Yes	36	32.7%
	No	74	67.3%
2. Oral health-related variables			
Toothbrushing Frequency	Once/day	73	66.4%
	>2 or more/per day	37	33.6%
Oral disinfection (use of antiseptic mouthwash)	Yes	23	20.9%
	No	87	79.1%
Oral disease (gingivitis, periodontitis, and tooth loss due to periodontitis)	Yes	1	0.9%
	No	109	99.1%
3. Scores	Plaque score	Gingival score	
Mean	1.59	1.68	
Median	2.00	2.00	
Std. Deviation	0.63	0.60	
Range	3.00	2.00	
Minimum	0.00	1.00	
Maximum	3.00	3.00	

**Table 3:** Pregnancy-related variables and bacterial species (*n* = 110).

Variables		*n*	%
Pregnancy frequency	Nonpregnant	20	18.2%
	First pregnancy	25	22.7%
	Two or more pregnancies	65	59.1%
Pregnancy trimester	Nonpregnant	20	18.2%
	First trimester	18	16.4%
	Second trimester	35	31.8%
	Third trimester	37	33.6%
Bacterial species	LF	26	23.6%
	LA	4	3.6%
	LG	1	0.9%
	AN	40	36.4%
	AO	3	2.7%
	PD	6	5.5%
	CL	2	1.8%
	VL	11	10.0%
	BB	6	5.5%
	LP	1	0.9%
	UO	10	9.1%
Gram staining	Gram-positive	83	75.5%
	Gram-negative	20	18.2%
	Unknown	7	6.3%

*Lactobacillus fermentum* (LF), *Lactobacillus acidophilus* (LA), *Lactobacillus gasseri* (LG), *Actinomyces naeslundii* (AN), *Actinomyces odontolyticus* (AO), *Parabacteroides distasonis* (PD), *Clostridium* group (CL), *Veillonella* (VL), *Bifidobacterium* spp. (BB), *Lactobacillus plantarum* (LP), *Unidentified organism* (UO).

**Table 4:** Association between pregnancy status and personal characteristics.

Variables		Pregnant (*n* = 90)		Nonpregnant (*n* = 20)		Total (*n* = 110)		*p* *
		n	%	n	%	n	%	
Age (years)	18–25	10	11.1	4	20.0	14	12.7	0.281
	26–40	80	88.9	16	80.0	96	87.3	
Weight (kg)	<60	3	3.3	0	0.0	3	2.7	0.408
	61–100	87	96.7	20	100.0	107	97.3	
Height (cm)	140–180	87	96.7	18	90.0	105	95.5	0.195
	180–200	3	3.3	2	10.0	5	4.5	
Education	School	42	46.7	4	20.0	46	41.8	0.029 *
	University	48	53.3	16	80.0	64	58.2	
Brushing frequency	Once/day	67	74.4	6	30.0	73	66.4	<0.001
	≥2/day	23	25.6	14	70.0	37	33.6	
Oral disinfection (use of antiseptic mouthwash)	Yes	18	20.0	5	25.0	23	20.9	0.619
	No	72	80.0	15	75.0	87	79.1	
Sugar drinks	Yes	27	30.0	9	45.0	36	32.7	0.196
	No	63	70.0	11	55.0	74	67.3	

* Chi-square test.

**Table 5:** Different bacterial species in pregnant and nonpregnant women.

Bacterial Species	Pregnant (*n* = 90)		Nonpregnant (*n* = 20)		Total (*n* = 110)		*p* §
	*n*	%	*n*	%	*n*	%	
LF	17	18.9	9	45.0	26	23.6	**<0.001**
LA	3	3.3	1	5.0	4	3.6	
LG	1	1.1	0	0.0	1	0.9	
AN	40	44.4	0	0.0	40	36.4	
AO	2	2.2	1	5.0	3	2.7	
PD	6	6.7	0	0.0	6	5.5	
CL	1	1.1	1	5.0	2	1.8	
VL	11	12.2	0	0.0	11	10.0	
BB	4	4.4	2	10.0	6	5.5	
LP	1	1.1	0	0.0	1	0.9	
UO	4	4.4	6	30.0	10	9.1	

*Lactobacillus fermentum* (LF), *Lactobacillus acidophilus* (LA), *Lactobacillus gasseri* (LG), *Actinomyces naeslundii* (AN), *Actinomyces odontolyticus* (AO), *Parabacteroides distasonis* (PD), *Clostridium* group (CL), *Veillonella* (VL), *Bifidobacterium* spp. (BB), *Lactobacillus plantarum* (LP), *Unidentified organism* (UO). § Fisher’s exact test.

## Data Availability

All the data are contained within the article.
